# p32 regulates glycometabolism and TCA cycle to inhibit ccRCC progression via copper-induced DLAT lipoylation oligomerization

**DOI:** 10.7150/ijbs.84399

**Published:** 2024-01-01

**Authors:** Shaoping Tian, Rui Wang, Yiting Wang, Ruibing Chen, Tianyu Lin, Xuesong Xiao, Xinyu Liu, Justin Eze Ideozu, Hua Geng, Yong Wang, Dan Yue

**Affiliations:** 1 Department of Microbiology, School of Medical Laboratory, Tianjin Medical University, Tianjin 300203, China.; 2 Department of Urology, Tianjin Institute of Urology, The Second Hospital of Tianjin Medical University, Tianjin 300211, China.; 3 Department of Clinical Laboratory, Tianjin Children's Hospital/Tianjin University Children's Hospital, Tianjin 300134, China.; 4 School of Pharmaceutical Science and Technology, Tianjin University, Tianjin 300072, China.; 5 Genomic Medicine, Genomic Research Center, AbbVie, North Chicago, IL 60064, USA.; 6 Department of Pediatrics, University of Illinois at Chicago, Chicago, IL, USA.

**Keywords:** Clear cell renal cell carcinoma, p32, glycometabolism, DLAT, tricarboxylic acid cycle, Copper

## Abstract

A key player in mitochondrial respiration, p32, often referred to as C1QBP, is mostly found in the mitochondrial matrix. Previously, we showed that p32 interacts with DLAT in the mitochondria. Here, we found that p32 expression was reduced in ccRCC and suppressed progression and metastasis in ccRCC animal models. We observed that increasing p32 expression led to an increase in oxidative phosphorylation by interacting with DLAT, thus, regulating the activation of the pyruvate dehydrogenase complex (PDHc). Mechanistically, reduced p32 expression, in concert with DLAT, suppresses PDHc activity and the TCA cycle. Furthermore, our research discovered that p32 has a direct binding affinity for copper, facilitating the copper-induced oligomerization of lipo-DLAT specifically in ccRCC cells. This finding reveals an innovative function of the p32/DLAT/copper complex in regulating glycometabolism and the TCA cycle in ccRCC. Importantly, our research provides important new understandings of the underlying molecular processes causing the abnormal mitochondrial metabolism linked to this cancer.

## Introduction

With over 400,000 new instances of cancer diagnosed each year and a death rate of over 175,000, kidney cancer is the third most common genitourinary malignancy worldwide 1. Three main histological subtypes of renal cell carcinoma (RCC) have been identified: papillary (15% to 20%), chromophobe (5%) and clear cell (75%) 2. The propensity for clear cell renal cell carcinoma (ccRCC) to be identified at an advanced stage significantly reduces disease-specific survival 3, 4. The tricarboxylic acid cycle (TCA cycle), glucose metabolism, and fatty acid metabolism are all altered in ccRCC, suggesting a reprogramming of these metabolic processes 5-7. As a result, it is aptly regarded as a metabolic disease. Mitochondria dysfunction is a well-documented feature of many metabolic diseases, including ccRCC. It has been proposed that ccRCC encourages pyruvate to be converted to lactate, which is then secreted via mitochondrial metabolism as opposed to glycolysis. The metabolic shift—also known as the Warburg effect or aerobic glycolysis—is a characteristic that sets many malignancies, including ccRCC, apart 8.

A complicated set of metabolic activities known as glycolysis turns glucose into pyruvate. Pyruvate is then converted to acetyl-CoA by the PDHc in the majority of differentiated human tissues, allowing it to join the mitochondrial TCA cycle. This mechanism is essential to the production of ATP via oxidative phosphorylation (OXPHOS), the cell's main source of energy 9. The Warburg effect, or increased glycolysis in tumor cells, is accompanied with increased glutamine use to maintain the operation of the TCA cycle 10, 11. Ultimately, the recognition of mitochondrial dysfunction and aerobic glycolysis as prominent characteristics of cancer has gained widespread acceptance 12. By generating oncometabolites to preserve the malignant phenotype and providing necessary metabolites for macromolecular synthesis, mitochondrial metabolism maintains tumor anabolism 13. Numerous clinical studies are being conducted to explore the therapeutic potential of focusing on mitochondrial metabolism as a novel strategy for treating cancer 14, 15. Many early clinical trials, including those evaluating the Telaglenastat (CB-839), a glutaminase inhibitor, combined with other therapies, and CPI-613, a mitochondrial inhibitor, combined with other therapies, have yielded positive outcomes as cancer therapies 16-20. Thus, it is conceivable that targeting the mitochondrial TCA cycle could be efficacious as a cancer therapy.

p32, also known as C1QBP, HABP1, and gC1qR, exhibits predominant localization within the mitochondrial matrix, while its expression is comparatively lower in the cytoplasm, nucleus, and cell surface. This subcellular distribution suggests that p32 primarily functions within the mitochondria, potentially participating in various mitochondrial processes and interactions 21. The respiratory chain complex in neoplastic cells depends on p32, a pleiotropic chaperone protein, for its integrity and activity. It is also necessary for the formation of mitochondrial ribosome complexes 22, 23. p32 is highly expressed in a wide range of cancer types and is essential for metastasis and chemotaxis. Nonetheless, some data indicate that p32 may be downregulated in human cervical squamous cell cancer 24. In our previous study, we presented data in favor of p32's tissue-specific expression pattern and tumor-suppressive function in RCC. Our findings revealed that p32 exerts its regulatory effects on RCC metastasis through modulation of the GSK3/β-Catenin/L1CAM signaling pathway 25. Despite p32 commonly being referred to as a mitochondrial protein, the exact mechanisms underlying its contribution to mitochondrial dysfunction in ccRCC remain unclear.

Within the mitochondrial matrix lies a multi-enzyme complex known as the PDHc. It is essential for the effective linkage between glycolysis and the TCA cycle because it catalyzes the pyruvate to acetyl-Co conversion 26. The TCA cycle and glycolysis are connected by the PDHc, a trimeric enzyme complex that is necessary for the conversion of pyruvate to acetyl-CoA. Dihydrolipoyl transacetylase (E2), pyruvate dehydrogenase (E1), and dihydrolipoyl dehydrogenase (E3) are its three enzyme constituents. For this complex to be active and operate well, its integrity is essential 27. Although the exact mechanism behind PDHc activity maintenance in cancer is yet unknown, PDHc activation is inhibited by DLAT lipoylation and PDHA phosphorylation 28. Previously, we demonstrated that p32 interacts with DLAT in the mitochondria 29. However, the function of p32-DLAT in ccRCC remains unexplored.

The purpose of this work was to clarify p32's roles in the mitochondria of ccRCC. This involves p32 regulation of cellular energy metabolism and regulation of the TCA cycle to promote ccRCC progression via cooper-induced lipo-DLAT oligomerization. Thus, we have characterized a potentially novel mechanism that could be targeted for metabolic cancer therapy.

## Materials and methods

### Xenotransplantation

Animal studies were carried out in accordance with the National Institutes of Health's handbook for the care and use of laboratory animals (NIH Publications No. 8023, updated 1978) and the ARRIVE criteria.

Six BALB/c nude mice, each aged between six and eight weeks, were divided into two groups at random. Matrigel was combined 1:1 with a total of 1×10^6^ ACHN cells that had been stably transfected with either luciferase-labeled pCDH or pCDH-p32. The mixture was then collected and centrifuged for 5 to 8 minutes at 2,000 rpm. Anesthetized nude mice were given subrenally injected cells to create orthotopic xenograft models. Following an eight-week duration, every mouse received an intravenous injection of 30 μg of *in vivo* grade Luciferin (VivoGlo, P1042). To detect primary tumors and metastases in the liver and lung, bioluminescence imaging was performed using the live IVIS imaging system (Perkin Elmer, USA). The mice were then put to sleep, and the livers, lungs, and main tumors were surgically removed. The weight of the matching right kidney was subtracted to get the tumor weights. After being removed, the tissues were fixed in 4% formaldehyde, embedded in paraffin, and cut into consecutive 5.0 μm slices. Hematoxylin and eosin (H&E) stained sections were used for histologic investigation in order to assess tumor spread.

### Cell culture and transfection

The American Type Culture Collection provided the cell lines (HEK-293T, ACHN, and 786-O), which were then cultivated in accordance with the manufacturer's instructions. While the 786-O and HEK-293T cells were cultivated in DMEM (Biological Industries, 06-1055-57-1ACS), the ACHN cells were grown in MEM (Biological Industries, 01-042-1ACS). 10% FBS (Biological Industries, 04-001-1A-CS) and 1% PS (Biological Industries, 03-031-1B) were added to both medium as supplements. After being cleaned with PBS, the medium was changed to just DMEM or MEM for starving. Every cell line was cultured at 37 °C with 5% CO_2_.

Following the manufacturer's instructions, ACHN and 786-O cells were transfected with three separate DLAT siRNA oligonucleotides and non-targeting sequences (negative controls) from Sangon Biotech, China, using lipofectamine 2000 (Invitrogen, 11668-019). In the rescue tests, 8 μL of the necessary siRNA (20 μM) was transfected into p32-overexpressing cells that were grown on 6-well plates at a density of 70%-80%. After transfection, the siRNA-containing Opti-MEM medium (Gibco, 31985070) was changed every four to six hours. Following transfection, total RNA and protein were extracted 24 and 48 hours later, respectively. Both western blot and quantitative real-time polymerase chain reaction (qRT-PCR) methods were used to evaluate the knockdown of gene expression. Table S1 contains the DLAT siRNA sequences.

To investigate the interaction site between p32 and DLAT, we designed and purchased expression vectors for different domains of p32 tagged with FLAG from General Biosystems (Anhui, China). The domains included p32 full length (amino acids 1-282), p32 without the transit-peptide domain (amino acids 74-282), C-terminal p32 (amino acids 175-282), and N-terminal p32 (amino acids 74-174). Each experimental group was plated with HEK-293T cells on a 10-cm plate at a density of 60%-70%. Lipo2000 was employed for transient transfection of the expression vectors, and the amount of transfection plasmid was adjusted based on transfection efficiency. After 4-6 hours of transfection, 10% fetal bovine serum was added to DMEM, which was used in place of the plasmid-containing media. Domain immunoprecipitation experiments were conducted 48 hours post-transfection.

The development and identification of a lentiviral interference vector that targets p32 has already been finished by our group 30. HEK-293T cells were transfected with lentiviral vectors, such as pLKO.1-Scr (control), pLKO.1-shp32 (p32 knockdown), pCDH (control), and pCDH-p32 (p32 overexpression), along with lentivirus packaging plasmids (psPAX2 and pMD2G), using Lipo2000, in order to produce stable clones with p32 knockdown or overexpression. The transfection solution containing plasmids was changed four to six hours after transfection. The lentivirus particles were collected, centrifuged, and filtered through a 0.45 μm filter following a 48-hour transfection period. Next, 786-O and ACHN cells were infected with lentivirus particles independently over 24 hours. After 48 hours, the cells were selected using 2 μg/ml puromycin (Sangon Biotech, A610593) for a period of 10 days. The cells were then kept alive with 1 μg/ml puromycin. Using western blot and qRT-PCR, the expression of p32 in the stable cell lines was ascertained.

### RNA extraction, reverse transcription and quantitative real-time PCR

Trizol reagent (Ambion, 15596018) was utilized to extract total RNA. Next, cDNA synthesis was performed on 1 μg of RNA using the HiScript Q RT SuperMix for qPCR (Vazyme, R123). Following the manufacturer's instructions, the ChamQ Universal SYBR qPCR Master Mix (Vazyme, Q711) was used to perform quantitative RT-PCR. Sangon Biotech manufactured the qRT-PCR primers, which are provided in Table S2. The target gene's expression levels were standardized to GAPDH, and the 2^-ΔΔCt^ technique was employed for data analysis.

### Immunoprecipitation and immunoblotting

Cells grown in 10-cm dishes were lysed on ice using IP buffer (40 mM Tris-Cl, 120 mM NaCl, 1% Triton X-100), phosphatase inhibitors (1 mM NaF, 1 mM Na_3_VO_4_), and a protease inhibitor cocktail (Roche, 04693159001), for 30 minutes, to perform immunoprecipitation. Following the cell lysis, centrifugation was carried out at 12,000 rpm for 30 minutes to obtain clear lysates. The total protein concentrations of the cell lysates were determined using the BCA protein assay (Thermo Scientific, 23225). For the purpose of immunoprecipitation, the cell lysates were incubated for 6 hours at 4°C with 4 μg of a rabbit anti-p32 polyclonal antibody (Abcam, ab101267) and Dynabeads Protein A (Invitrogen, 10001D). As a negative control, Normal Rabbit IgG (Cell Signaling Technology, 2729S, 4 μg) was used. The antibody-bead complexes were then co-incubated with the cell lysates at 4°C for an additional night after the first incubation. Subsequently, the immunoprecipitates were resuspended in 2×SDS loading buffer after the beads had been cleaned five times using IP buffer. Western blotting was done after p32 and its associated proteins were isolated using antibody-conjugated beads.

The cells were lysed in accordance with the previously specified methodology for the p32 domain immunoprecipitation after the p32 domain plasmids were transfected in a 10-cm plate for every experimental group. The cell lysates were then incubated at 4°C for a whole night for immune-precipitation using Anti-DYKDDDDK Magnetic Agarose (Thermo Scientific, A36797). Following five IP buffer washes, the beads were boiled in 2×SDS loading buffer and subjected to western blotting analysis.

The cells were lysed on ice in SDS lysis solution (containing 60 mM Tris-HCl [pH 6.8], 2% SDS, and 10% glycerol) supplemented with a protease inhibitor cocktail before being subjected to two rounds of ice-cold PBS washings in preparation for immunoblotting. After sonication to disrupt the proteins, the lysates were collected into centrifuge tubes and boiling at 97°C for 10 minutes was used to denature them. The BCA protein assay was then used to calculate the total protein content in the cell lysate solution. Protein lysates were not boiled or sonicated, and no reducing reagents were added to the loading buffer in order to create the non-reducing protein indicator. Protein samples in equal quantities were electrophoretically resolved on an 8-10% gel, and the resulting membranes were subsequently transferred to polyvinylidene difluoride (Millipore, IPVH00010). After blocking the membranes with 5% milk in TBST, primary antibodies and HRP-conjugated secondary antibodies (Affinity, S0001, S0002, 1:3000) were used to probe the membranes. For exposure, standard ECL (Affinity, KF003) was utilized, and a chemiluminescent machine (Bio-Rad, USA) was employed to capture the blot image. p32 (Abcam, ab101267, 1:3000); DLAT (Cell Signaling Technology, 12362S, 1:2000); LDHA (Abcam, ab101562, 1:3000); PKM2 (Cell Signaling Technology, 4053T, 1:2000); DYKDDDDK Tag (Cell Signaling Technology, 86861S, 1:3000); Lipoic Acid (Abcam, ab58724, 1:3000); and β-actin (Affinity, T0022, 1:3000) for loading control were employed in this investigation.

### Clinical specimens and online data collection

Along with 75 formalin-fixed and paraffin-embedded primary ccRCC specimens that contained both tumor and paired neighboring normal tissues, a total of 30 paired fresh ccRCC tissues and their matching adjacent normal tissues were obtained. The specimens were from Tianjin Medical University's Second Hospital. No adjuvant therapy was administered either before or after the radical or partial nephrectomy performed on any of the study's subjects. We gathered the clinical characteristics of the patients, including T stage, Fuhrman grade, tumor size, age, and gender. The ethics committee approved and the patients gave their informed consent for the collection of tissue samples and clinical data. Every specimen underwent histological testing in compliance with the American Joint Commission on Cancer TNM staging criteria, and the tumor was independently rated by two pathologists. The Tianjin Medical University Institutional Review Board approved this study, which was carried out in conformity with the Declaration of Helsinki's tenets. Before being included in this study, every patient gave their informed permission.

The UALCAN database is open and available to the public at http://ualcan.path.uab.edu/. We used the TCGA analysis module for mRNA level analysis and the CPTAC analysis module to look at the differential expression of p32 at the protein level. The TCGA database (https://portal.gdc.cancer.gov/) included the RNA sequencing data for 539 KIRC tissues and 72 para-tumor tissues, as well as the relevant clinicopathological data of the patients. For additional analysis, the transcript per million (TPM) format was used to the RNA sequencing data in FPKM format. During the classification-based statistical analysis, data lacking clinical information were excluded, and missing values were filtered. Detailed information regarding the selected patients in the TCGA tumor proteomics datasets can be found in Table S3.

### Immunohistochemical staining

The same patients provided the ccRCC and the surrounding normal tissues, which have been previously reported 30. The tissue pieces were dewaxed and then heated for 20 minutes at 98°C in citrate buffer (pH 6.0) (Solarbio, C1010). 3% hydrogen peroxide was used for a 15-minute treatment to inhibit the endogenous peroxidase activity. The presence of these proteins was subsequently ascertained by following standard protocol and employing anti-p32 (Santa Cruz Biotechnology, sc-23884, diluted 1:50) or anti-DLAT (Santa Cruz Biotechnology, sc-365276, diluted 1:50) antibodies. Independently, two pathologists assessed the p32 and DLAT immunostaining levels. Using the previously outlined methodology, this assessment was based on the percentage of tumor cells exhibiting positive staining (stain area) and the staining's intensity 30. Based on the percentage of positive cells, the proportion of positive tumor cells was given a score between 0 and 5. One was assigned to a weak staining, two to a moderate staining, and zero to a negative staining. The tumor cell percentage and staining intensity ratings were added to determine the final staining score. The scores that were recorded were classified as either positive (4-7) or negative (0-3).

### Immunofluorescence staining

For a full day, cells were incubated at 37°C on coverslips in 12-well plates. Mito-Tracker Red CMXRos (Beyotime, C1049B, 300 nM) was utilized for staining mitochondria at 37℃ for 30 minutes, while Rhodamine B Hydrazide (Alfachem, A317536, 50 μM) was used to stain intracellular copper at 37℃ for 50 minutes, separately. After rinsing the cells with ice-cold PBS, they were fixed for 30 minutes with 4% paraformaldehyde (PFA). The cells were permeabilized by incubating them in PBS with 0.2% Triton X-100 (Solarbio, T8200) for a duration of 10 minutes. The cells were then blocked for an hour at room temperature using 3% BSA in PBS. Subsequently, the cells were stained with Alexa Fluor 488 AffiniPure Goat Anti-Rabbit IgG (Abbkine, A23220, 1:100) and Alexa Fluor 647 AffiniPure Goat Anti-Mouse IgG (YEASEN, 33213ES60, 1:100) for one hour at room temperature after being incubated with the primary antibodies DLAT (Cell Signaling Technology, 12362S, 1:50) and p32 (Abcam, ab101267, 1:100) for the entire night at 4°C. Afterward, to see the nuclei, the coverslips were counter-stained with DAPI (Solarbio, C0060). The subcellular localization of DLAT proteins (violet), p32 proteins (green), mito-tracker (red), and RhB Hydrazide (red) was then examined using a confocal microscope (Zeiss Lsm800, Germany) at a magnification of ×630.

### Lactate production assay

The Lactic Acid Assay Kit (Keygentec, KGT023) was used to detect the cells in accordance with the manufacturer's instructions. A density of 2×10^5^ cells per well was used to seed adherent cells in a 12-well plate, followed by overnight culture. After being gathered into centrifuge tubes, the cells were again suspended in 150 μl of DDW. Prior to use, an enzyme working solution was prepared by mixing Buffer B and Buffer A at a volume ratio of 1:100. 0.02 ml of sample, 1 ml of enzyme working solution, and 0.2 ml of chromogenic reagent were added to each detection well. The mixture was thoroughly mixed and then incubated at 37℃ for 10 minutes to allow the reaction to take place. Following the addition of two milliliters of stop solution to the well, the mixture was well mixed, and a BioTek microplate reader was used to measure the absorbance at 530 nm. After that, the acquired absorbance values were adjusted for the concentration of protein.

### ATP level assay

Using an ATP test kit (Nanjing Jiancheng Bio, A095-1-1), ATP levels were measured in accordance with the manufacturer's instructions. One million cells were taken, mixed with 300 microliters of hot DDW, sonicated, and then incubated for ten minutes in a boiling water bath. Each test sample received 30 μl of the sample and 330 μl of the working solution after the cells had been mixed and vortexed for one minute. After thoroughly mixing, the mixture was incubated at 37°C for 30 minutes. Each tube was then filled with 50 μl of precipitant, well mixed, and centrifuged for five minutes at 4,000 rpm. After that, 300 μl of the supernatant was poured into a fresh centrifuge tube along with 500 μl of color development solution. Next, the mixture was stirred for two minutes. In each tube, add 500 μl of terminator and stir for five minutes. Using a BioTek microplate reader, the absorbance at 636 nm was measured and then normalized to the protein concentration.

### PDH activity assay

The Pyruvate Dehydrogenase Kit (Abcam, ab287837) was used to identify the cells in accordance with the manufacturer's instructions. For ten minutes on ice, 1×10^6^ cells were lysed using 100 μl PDH assay buffer. After 5 minutes at 4°C and 10,000 g of spinning, the supernatant was poured into a fresh centrifuge tube. Next, 2 μl of substrate buffer, 2 μl of developer buffer, 46 μl of assay buffer, and 50 μl of sample were added to each detection well. The absorbance at 450 nm was measured in kinetic mode for a duration of 10-60 minutes at 37℃ immediately upon initiation of the reaction. Subsequently, the obtained absorbance values were normalized to the protein concentration.

### Extracellular acidification rate (ECAR) and oxygen consumption rate (OCR) assay

The extracellular acidification rate (ECAR) and oxygen consumption rate (OCR) were measured using the Seahorse XFe24 extracellular flux analyzer (Agilent Technologies, USA), in accordance with the guidelines included in the manufacturer's protocol. The cells were plated on an XFe24 plate at a density of 2×10^4^ cells/well, and then incubated for the whole night. One hour prior to the assay, the media were replaced with XF media. To conduct the ECAR assay, XF media was used to dilute Glucose (Sigma, G7021), Oligomycin (MedChemExpress, HY-N6782), and 2-deoxy glucose (2-DG; Sigma, D8375). The cartridge was subsequently filled with these diluted chemicals at concentrations of 10 mM, 1 μM, and 50 mM, in that order. Oligomycin, FCCP (Absin, abs816229), Antimycin A (Biovision, 2247), and Rotenone (MedChemExpress, HY-B1766) were diluted in XF medium in order to conduct the OCR test. The cartridge was then filled with these diluted substances at concentrations of 1 μM, 2 μM, 0.5 μM, and 0.5 μM, in that order.

### Cell proliferation assay

The cells were cultivated using the previously reported protocol after being plated in 96-well plates at a density of 1×10^4^ per well. Cell proliferation was assessed using the Cell Counting Kit-8 (CCK-8; Biosharp, BS350B) at 24, 48, 72, and 96 hours after seeding, in accordance with the manufacturer's recommendations. To sum up, 10 μL of the CCK-8 solution was put into each well of the plate, and it was then incubated for two hours at 37 °C. For every well, the absorbance at 450 nm was measured using a microplate reader.

### Cell migration assay

Transwell chambers (LABSELECT, 14342) with polyester membranes containing 8 μm holes were used to carry out the migration test. The bottom chamber was filled with media containing 10% FBS, while the top chamber was filled with ccRCC cells (5×10^5^ cells/chamber) in serum-free medium. The cells attached to the bottom side of the membrane were fixed and stained, while the cells on the top side of the membrane were removed after 4 hours of incubation at 37°C. Under a microscope, the numbers of cells were counted in five randomly selected areas.

### Copper affinity binding assay

Cu IDA Beads (Smart-Lifesciences, SA041005) and Ni IDA Beads (negative control; Smart-Lifesciences, SA052005) were subjected to washing and subsequently incubated with cell lysates at 4℃ overnight, following the manufacturer's recommended protocol. The beads were subjected to 3-5 washes with PBS, followed by elution with 2×loading buffer. Subsequently, the eluted sample was boiled at 97℃ and then centrifuged. The eluted proteins were then immunoblotted using the previously mentioned protocol after being exposed to SDS-PAGE analysis.

### Drug survival experiment

Plated in a 96-well plate, 5×10^4^ cells per well were grown according to the previous instructions. CuSO_4_ (Aladdin, C119000) and Elesclomol (Aladdin, E126032) were introduced at varying quantities in a 1:1 ratio to every well. 24 hours following treatment, cell survival was assessed using the manufacturer's technique as previously outlined using the Cell Counting Kit-8 (CCK-8; Biosharp, BS350B).

### Protein cross-linking

1×10^6^ cells were plated in each well of a 6-well plate, and the cells were treated with a gradient chemical for a whole day. Clean the cells with PBS twice after digestion and collection. To obtain final concentrations of 0.1 mM, a crosslinker, disuccinimidyl suberate (DSS; Solarbio, D9480), was applied. Tris buffer (pH 7.5) was added to the tubes and gently rotated for 30 minutes at room temperature. The reaction was then quenched for 15 minutes by adding Tris buffer at a final concentration of 50 mM. Cells were washed twice and cleavage protein according to the above method. Proteins were separated using 8% SDS-page, and crosslinked products were detected using antibodies on western blots.

### Transmission electron microscope experiment

The cells were digested and collected in a centrifuge tube for 3 minutes at 1000 rpm. After washing with PBS and centrifuging, the supernatant was removed. Slowly applied 2.5% glutaraldehyde fixing solution along the pipe wall, then left overnight at 4℃. Fix the previously fixed cell sample with osmic acid after three rinses. After fixation, rinse three times with buffer solution before dehydrating for 15 minutes with ethanol solution of varying concentrations. Following dehydration, the sample is treated for 20 minutes with pure acetone, followed by a combination of acetone and embedding agent (V/V=1/1), a transfer to a mixture of acetone and embedding agent (V/V=3/1), and an overnight treatment with pure embedding agent. The sample is obtained by heating the penetrated sample to 70°C for a certain amount of time and then embedding it. The samples were sectioned using an ultrathin sectioning machine into 70-90 nm slices. Following coloring with lead citrate and uranium acetate solutions, the slices were dried and viewed under a transmission electron microscope.

### Statistical analysis

The student's *t*-test was used for all statistical analysis in the study to compare variables between two groups, and the ANOVA test was used for comparisons between multiple groups. The Chi-square test was used to statistically analyze the relationships between clinic-pathological features and protein expression. The associations between the expressions of p32 and DLAT were investigated using the Spearman test. The mean ± SEM was given as the value. Disparities were deemed statistically significant when their *P < 0.05 significance level was met. We used GraphPad Prism 8.0 for statistical analysis.

## Results

### p32 expression is low in ccRCC and correlated with tumor metastasis and prognosis

**Figure 1:**
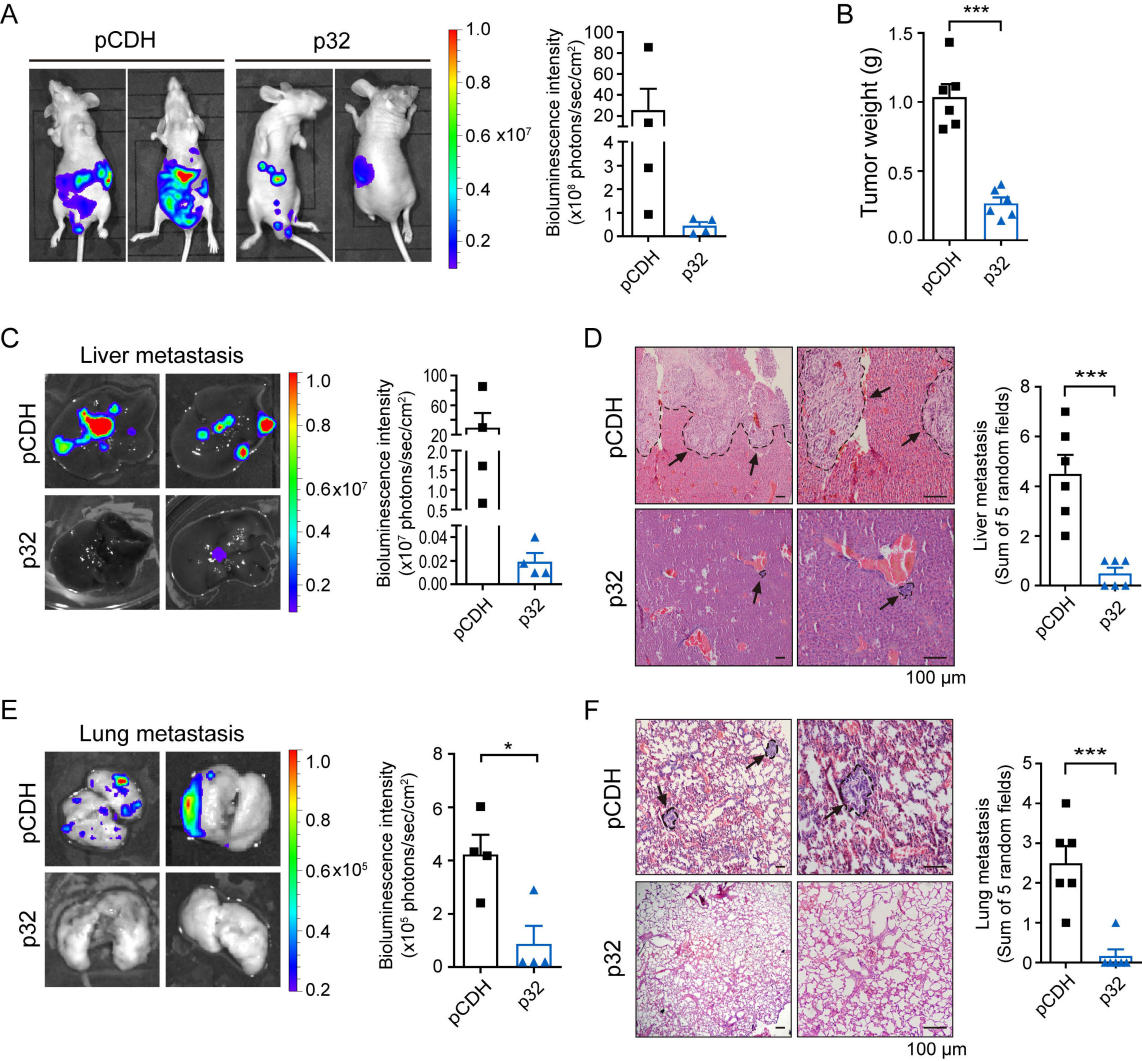
p32 inhibits proliferation and metastasis of ccRCC *in vivo*. **(A)** Luciferase-expressing p32-overexpression and pCDH-control ACHN cells were injected into the left sub-renal capsule of nude mice and then raised for 8 weeks (n=6). After anesthetized nude mice and injected fluorescein intraperitoneally for 10 minutes, luminescence intensity derived from tumors was measured by IVIS imaging system, and representative bioluminescence images were shown (left panel). Right panel showed quantitative analysis of bioluminescence intensity in nude mice (n=4, *P*=0.256). **(B)** Statistical analysis of in situ tumor weight, expressed as the weight of the left minus the corresponding right kidney of nude mice (n=6, *P*<0.001). **(C)** Liver and **(E)** lung of nude mice were isolated, and bioluminescence intensity was detected by IVIS imaging system (left panel). Right panel showed quantitative analysis of liver and lung bioluminescence intensity (n=4, Liver *P*=0.1901, Lung *P*=0.0153). **(D)** Liver and **(F)** lung metastatic foci were observed after H&E staining, indicated by black arrows (left panel). Metastatic foci in the liver and lung were quantified and compared using five random microscopic views (right panel, n=6, Live *P*=0.0005, Lung *P*=0.0005). Data represent the mean ± SEM of three independent experiments. *P*-values were determined by unpaired two-tailed Student's *t*-test.

To identify the p32 function, we used the UALCAN database 31 to investigate pan-cancer gene expression of p32 in ccRCC. Variable disparities in the p32 gene expression patterns were observed between the matched normal tissues and the tumor. As compared to normal tissues, bladder, breast, colon, liver, prostate, and lung cancers showed higher expression of *p32*, whereas kidney, thyroid, pheochromocytoma, and paraganglioma tumors showed lower expression of *p32* (Figure S1A). The UALCAN was used to analyze the TCGA tumor proteome datasets, and the results indicated that the considerably lower expression of p32 in ccRCC is statistically more significant than in other tumor types (Figure S1B). TCGA analysis revealed decreased p32 mRNA and protein expression in ccRCC, as we have previously shown and confirmed with clinical samples 30, 32, 33. Next, we looked at p32's potential role in ccRCC tumor development and metastasis. We investigated the impact of injecting ACHN cells orthotopically into the left kidney of mice. Our data revealed remarkable tumor growth in these animals over 8 weeks. However, mice implanted orthotopically with p32 overexpression ACHN cells exhibited a much smaller tumor size (Figure 1A). When compared to the pCDH-control group, the weight of kidney tumors was considerably lower in the p32 overexpression group (Figure 1B). Meanwhile, bioluminescence intensity was used to evaluate the liver and lung metastasis before mice were sacrificed. In the p32 overexpression group, in the liver and lungs, we discovered that bioluminescence signals were noticeably lower than in the control group (Figure 1C, E). H&E staining also showed fewer liver and lung metastases in the p32 overexpression group (Figure 1D, F). Taken together, results indicated that p32 has a diminished expression in ccRCC and negatively regulates ccRCC metastasis *in vivo*.

### p32 and DLAT are correlated in ccRCC tissues

**Figure 2:**
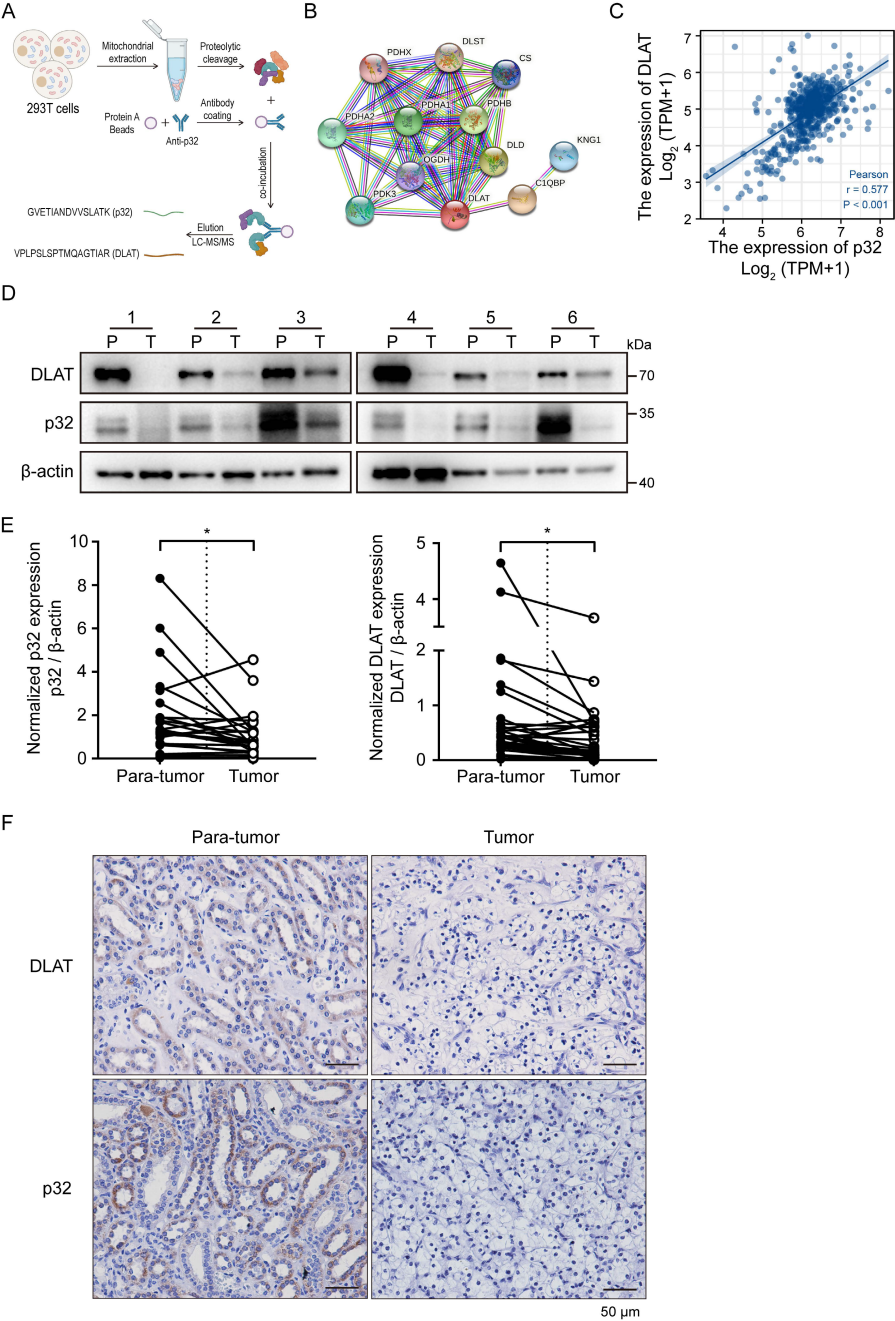
Correlation between p32 and DLAT at the clinical tissue sample levels. **(A)** Schematic diagram showed the identification of proteins interacting with p32 in mitochondria by mass spectrometry. **(B)** A network map of proteins predicted to interact with p32 using the STRING database (https://cn.string-db.org/). **(C)** Correlation analysis between the expression levels of p32 and DLAT in the TCGA-KIRC data set (n=539, r=0.577, *P*<0.001). **(D)** Representative immunoblot images of p32 and DLAT protein levels in para-tumor (P) and tumor (T) tissues of 6 primary renal cell carcinoma patients. **(E)** The expression of p32 (left panel) and DLAT (right panel) was normalized with β-actin and quantified by Image J software in 30 primary RCC patients. The analysis was used paired two-tailed Student's *t*-test, n=30, p32 *P*=0.0146, DLAT *P*=0.0129, respectively. **(F)** Immunohistochemistry staining was used to examine the expression of p32 and DLAT in a study cohort consisting of 75 pairs of adjacent normal kidney tissues (Para-tumor) and RCC tissues (Tumor). Representative images were provided, where the presence of brown signals indicated positive staining.

RCCs are typically categorized as metabolic diseases 6, including ccRCC. Somatic mutations in metabolic pathway genes, including those pertaining to regulatory genes connected to fatty acid metabolism, aerobic glycolysis, and tryptophan glutamine use, are the hallmark of RCC 7. Mitochondria are recognized as highly dynamic organelles responsible for orchestrating essential cellular metabolic pathways and bioenergetics processes 34. p32 is a toroidal (doughnut-shaped) protein that exhibits a high degree of evolutionary conservation across eukaryotes. It is primarily found within the mitochondria, representing its canonical subcellular localization 35. On this basis, we carried out proteomics analysis to identify p32 binding proteins in the mitochondria. We performed p32 immunoprecipitation (IP) using p32 antibody with mitochondrial extracts from HEK-293T cells 29. Dihydrolipoyllysine-residue acetyltransferase component of pyruvate dehydrogenase complex (DLAT) was shown to be an abundant protein in p32-IP by mass spectrometry analysis (Figure 2A). An online database called Search Tool for the Retrieval of Interacting Genes (STRING) 36 provided additional confirmation of the relationships between p32 and DLAT (Figure 2B). The kidney renal clear cell carcinoma (KIRC) dataset in TCGA was used for Spearman's rank correlation analysis, which showed a link between the mRNA expression levels of *p32* and DLAT (Figure 2C). Together, these results demonstrated that p32 potentially interacts with DLAT.

The mitochondrial protein DLAT is essential for the metabolism of glucose because it forms the core of the PDHc. The enzyme complex PDHc is in charge of catalyzing the change from pyruvate to acetyl-CoA. The TCA cycle facilitates the production of acetyl-CoA, which produces energy 28. We initially used UALCAN to measure the expression of *p32* and *DLAT* in ccRCC at the mRNA level in order to identify any potential changes to these genes in the cell. When ccRCC was compared to nearby normal tissues, bioinformatic analysis showed that the expression levels of both *p32* and *DLAT* mRNA were downregulated (Figure S1C). We also confirmed the mRNA expression of *DLAT* and *p32* in several patient subgroups with ccRCC. In comparison to the healthy controls, our results showed that ccRCC patients had lower levels of *p32* mRNA expression. The *DLAT* expression analysis produced comparable outcomes (Figure S1D-F). Prognostic analyses indicated that low expression of both *p32* and *DLAT* was significantly correlated with overall and disease-specific survivals in TCGA cohort (Figure S1G-J). In order to confirm the *p32* and *DLAT* expression levels in ccRCC tissues and matched nearby normal tissues, we further extracted protein. The findings showed that p32 and DLAT protein expression levels were both considerably lower in ccRCC tissues (Figure 2D, E). According to Table 1's statistical analysis, there was a positive correction in the expression of p32 and DLAT in ccRCC tissues. We then used immunohistochemistry (IHC) labeling for p32 and DLAT on ccRCC tumors in order to confirm the protein levels of these markers in ccRCC patients. In our investigation of 75 matched samples, we found that ccRCC tumors had considerably lower immunostaining intensities of both p32 and DLAT when compared to nearby normal tissue (Figure 2F). Additionally, statistical correlation analysis showed a positive link between p32 and DLAT (Table 2). It was also examined how p32 and DLAT expression related to the clinicopathologic characteristics of ccRCC. Table 3 shows a correlation between p32 expression and the tumor's T stage and Fuhrman grade. Together, our results established a positive correlation between p32 and DLAT expression in ccRCC, and their expression was associated with patient progression and survival.

### p32 promotes glucose metabolism of renal cancer cells towards OXPHOS rather than glycolysis

**Figure 3:**
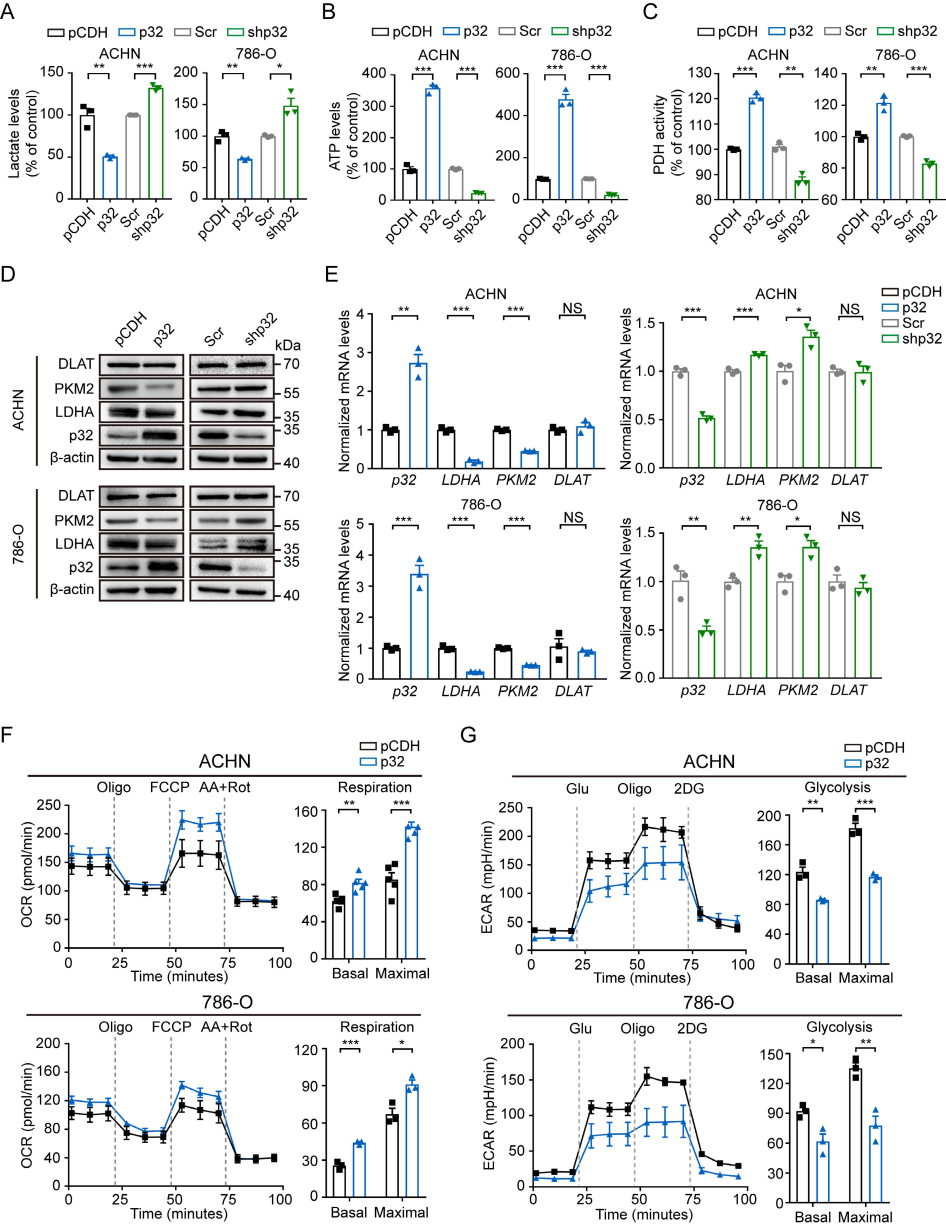
p32 regulates ccRCC glucose metabolism towards OXPHOS. **(A)** Lactate levels, **(B)** ATP levels and **(C)** PDH activity were examined with p32 overexpression and knockdown in ACHN (left panel) and 786-O cells (right panel) (n=3). **(D)** Representative immunoblot showed the level of glycolysis-related protein PKM2 and LDHA after p32 overexpression and knockdown in ACHN (upper panel) and 786-O cells (lower panel). **(E)** Quantitative RT-PCR analysis measured *LDHA* and *PKM2* relative mRNA levels in p32-overexpressed ACHN cells (upper left panel), p32-overexpressed 786-O cells (lower left panel), p32-knockdown ACHN cells (upper right panel) and p32-knockdown 786-O cells (lower right panel) (n=3). **(F)** p32-overexpressed ACHN cells (upper panel, n=5) and 786-O cells (lower panel, n=3) were seeded in Seahorse XFe24 cell culture plates and sequential treated with oligomycin (Oligo), FCCP and rotenone plus antimycin A (Rot/AA). Bar graphs showed quantified basal and maximal OCR. **(G)** Representative Seahorse analysis of extracellular acidification rate (ECAR) in p32-overexpressed ACHN cells (upper panel, n=3) and 786-O cells (lower panel, n=3). Bar graphs showed quantified basal and maximal ECAR. Data was analyzed by unpaired two-tailed Student's *t*-test. All error bars displayed mean ± SEM of three independent experiments. Statistically significant differences were indicated: *P<0.05, **P<0.01, ***P<0.001. NS: no significant difference.

ccRCC display distinct metabolic states in comparison to normal tissues but the molecular interplay between the altered metabolism and ccRCC progression or metastasis remain unclear. Using lentivirus-mediated p32 overexpression and knockdown, 786-O and ACHN cells were created to investigate the metabolic role of p32 in ccRCC cells. We initially looked at the creation of ATP and lactate, two key markers of the metabolic process in tumor cells, in order to ascertain if p32 is engaged in glucose metabolism. The ectopic expression of p32 decreased lactate generation and increased ATP production of 786-O and ACHN cells, as seen in Figures 3A and 3B, whereas p32 silencing increased lactate production and decreased ATP production of both cells. Our result showed that p32 was related to DLAT in ccRCC, which is an important subunit for PDHc. We then examined the endogenous cellular activity of PDH by overexpression or knockdown of p32 in ccRCC cells. PDH activity was diminished in p32 knockdown cells and increased in p32 overexpressing cells (Figure 3C). We further investaged the role of p32 in glycometabolism protein expression. Contrary to the increased expression of p32, there was a significant decrease of glycolysis enzymes, including LDHA and PKM2 in comparison to control samples in ccRCC cell lines (Figure 3D, E). Also, in 786-O and ACHN cells, the mitochondrial respiratory capacity (OCR) was assessed. It's interesting to note that cells overexpressing p32 had higher OCR values than the control group (Figure 3F). Next, we took ECAR measurements, which show how cells get acidified due to lactate. The findings showed that p32 overexpression reduced the ECAR value and could inhibit the production of lactate in ACHN and 786-O cells (Figure 3G). These results thus suggest that p32 is involved in the glucose metabolism of ccRCC cells and that it is preferentially involved in the metabolism of glucose towards OXPHOS rather than glycolysis.

### p32 promotes glucose metabolism via DLAT

**Figure 4:**
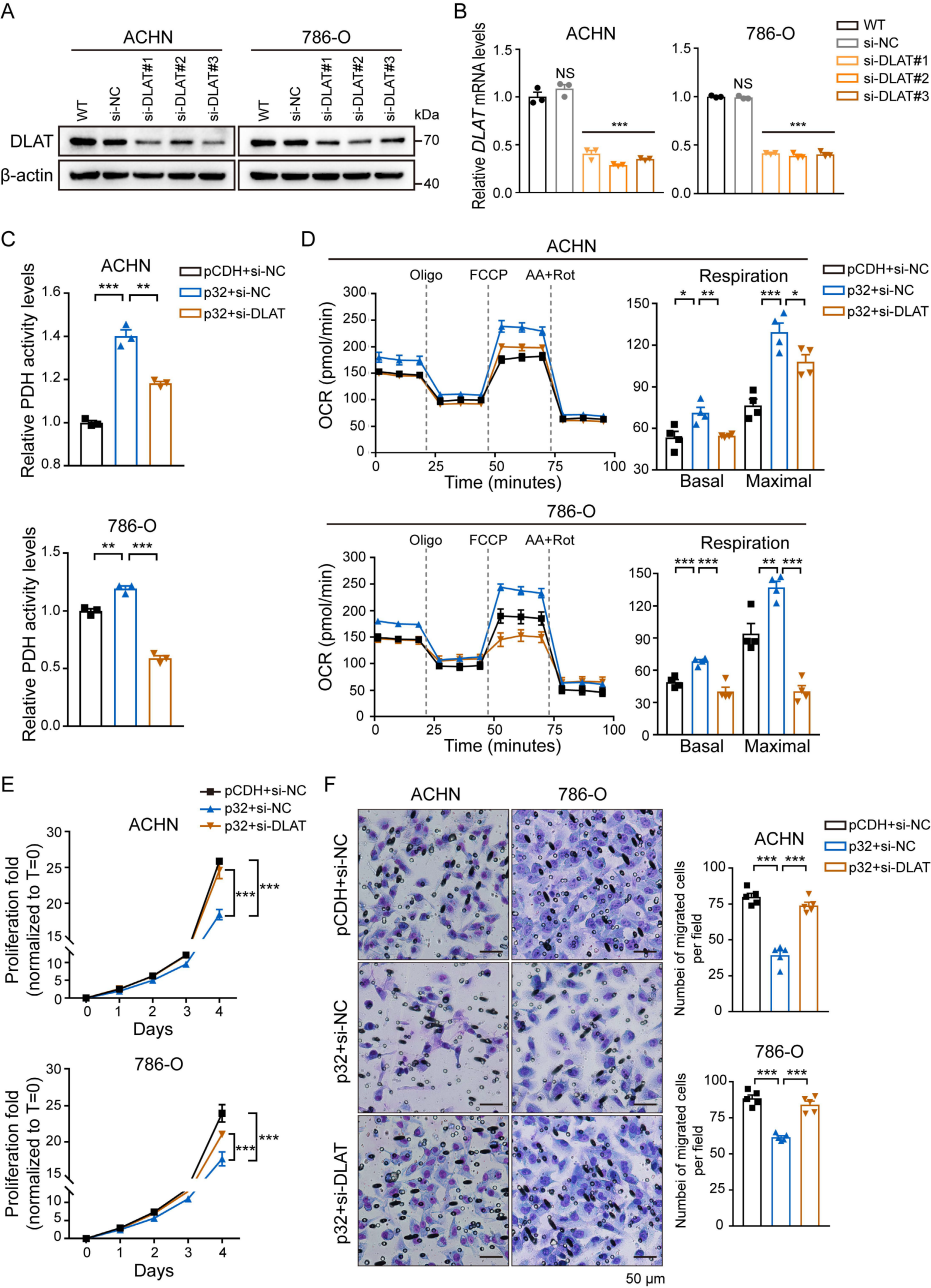
p32 regulates OXPHOS and tumor progression via DLAT in ccRCC. DLAT knockdown by using three independent DLAT siRNAs (si-DLAT#1, si-DLAT#2, si-DLAT#3) in ACHN and 786-O cells were evidenced by **(A)** western blot and **(B)** real-time PCR (n=3). **(C)** ACHN and 786-O cells were transfected with pCDH+si-NC, p32+si-NC, p32+si-DLAT, and then the level of PDH activity was measured after 48h (n=3). **(D)** Detection of intracellular oxygen consumption levels and quantification basal and maximal OCR after transfection (n=4). **(E)** Cell Counting Kit-8 reagent was used to measure the cell proliferation in treated ACHN and 786-O cells (n=3). **(F)** Left panels: the representative migration images of treated ACHN and 786-O cells, Right panels: Random five fields of view counting the number of cells per field. The results were the mean ± SEM of three independent experiments., *P*-values were determined by unpaired two-tailed Student's *t*-test. Statistically significant differences were indicated: *P<0.05, **P<0.01, ***P<0.001.

p32 does not possess any enzymatic domain, therefore, it may exert its function by interacting with other metabolic machineries. Given that PDH activity changed with the expression of p32 (Figure 3C) and the expression correlation between p32 and DLAT is strong in ccRCC tissues (Figure 2), we speculated that p32 regulates glucose metabolism through DLAT. siRNA-mediated knockout of DLAT was verified by protein and gene expression analysis. The outcomes demonstrated that in 786-O and ACHN cells, DLAT was effectively eliminated (Figure 4A, B). Furthermore, the enhanced impact of p32 overexpression on PDH activity in both 786-O and ACHN cells was further mitigated by temporary DLAT deletion (Figure 4C). In order to investigate DLAT reliance on p32 activity, we subjected p32 overexpression cells to DLAT deficiency and examined cellular OCR. Notably, the results showed that DLAT rescued basal respiration and maximal respiration induced by p32 overexpression (Figure 4D). Thus, we infer that p32 promotes glucose metabolism via DLAT. As expected, in 786-O and ACHN cells, DLAT deficiency dramatically restored the p32-suppressed proliferation and migration (Figure 4E, F). Taken together, these findings suggest p32 regulates ccRCC metastasis by modulating DLAT.

### p32 and DLAT colocalize and interact with peptides in renal cancer cells

**Figure 5:**
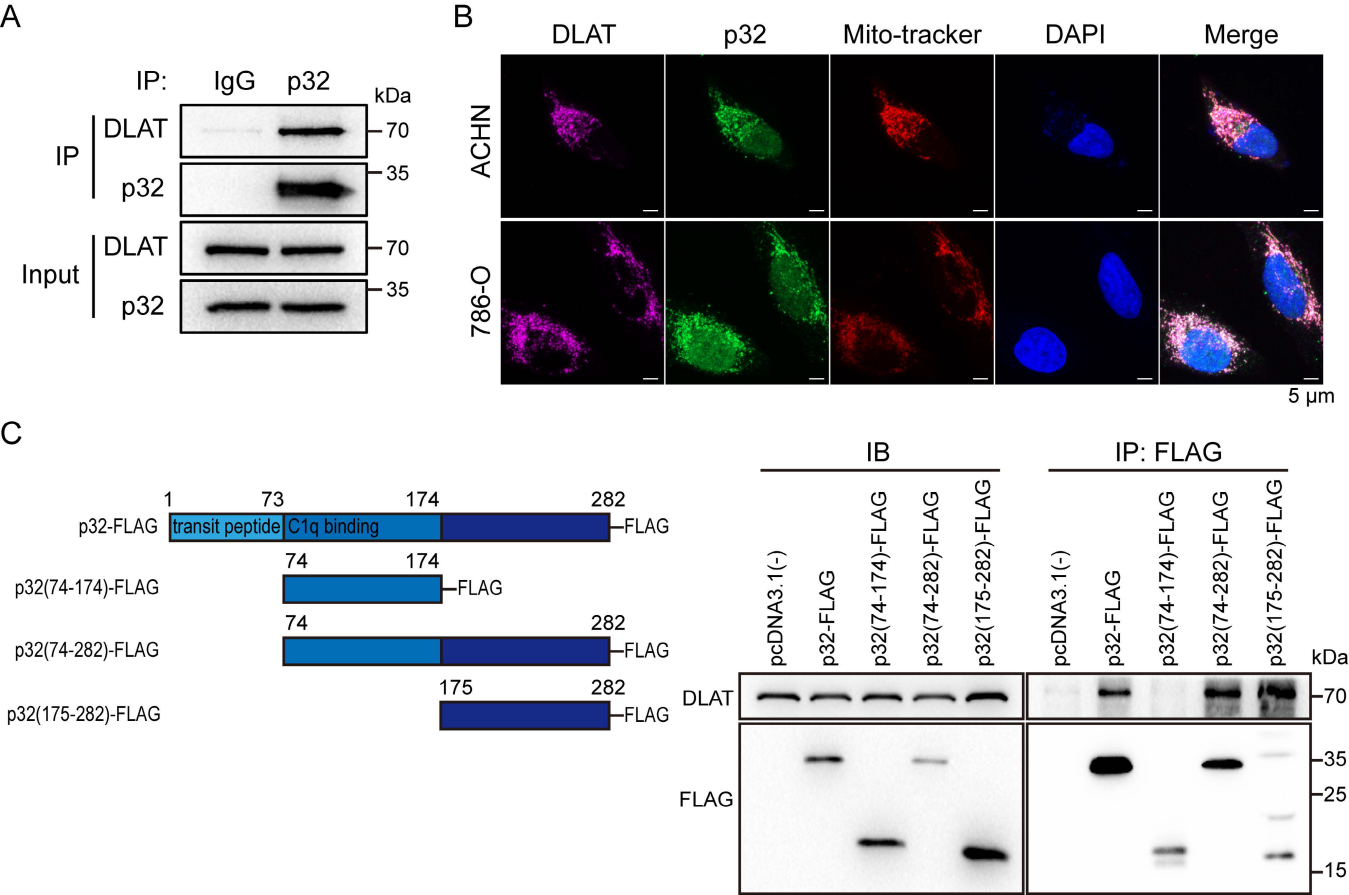
p32 and DLAT interactive with each other and colocalize. **(A)** Co-immunoprecipitation of DLAT with p32 in ACHN cells. **(B)** Fluorescence images of colocalization of p32, DLAT and mitochondria in ACHN (upper panel) and 786-O (lower panel). Mito-tracker probes labeled mitochondria via mitochondrial membrane potential. **(C)** Left panel: schematic representation of the FLAG-tagged p32 domain protein peptides. Right panel: Co-IP analysis showed interaction of DLAT with p32 (aa 175-282) after plasmid transfection into 293T cells for 48h.

p32 could not regulate the gene and protein expression of DLAT (Figure 3D, E), so we assumed that p32 interacts with DLAT to regulate ccRCC glucose metabolism. To validate the endogenous p32-DLAT interaction, a co-IP assay was conducted in ACHN cells. Immunoprecipitation was performed using an anti-p32 antibody, followed by detection of co-enriched DLAT through immunoblotting with an anti-DLAT antibod (Figure 5A). Co-staining p32 and DLAT with Mito-tracker showed that p32 colocalized with DLAT at the mitochondria (Figure 5B). To further identify the key structural domains for the interaction between p32 and DLAT, we constructed different truncated variants of p32, including the full-length p32 (FL), p32 without transit-peptide domain (aa 74-282), C-terminal p32 (aa 175-282) and N-terminal p32 (aa 74-174). We found that DLAT interacted with FL, p32 without transit-peptide domain and C-terminal p32, and that N-terminal p32 (missing 175-282 peptide domain) compromised the DLAT-p32 interaction (Figure 5C). These findings indicate that the p32 C-terminal (aa 175-282) is crucial for its physical interaction with DLAT. Altogether, our results demonstrate that p32 interacts with DLAT. And we discussed these functional analyses that N-terminal p32 reveal an invalid role owing to lost interaction of DLAT (Figure S2A, B).

### p32 promotes lipo-DLAT oligomerization via binding copper in renal cancer cells

**Figure 6:**
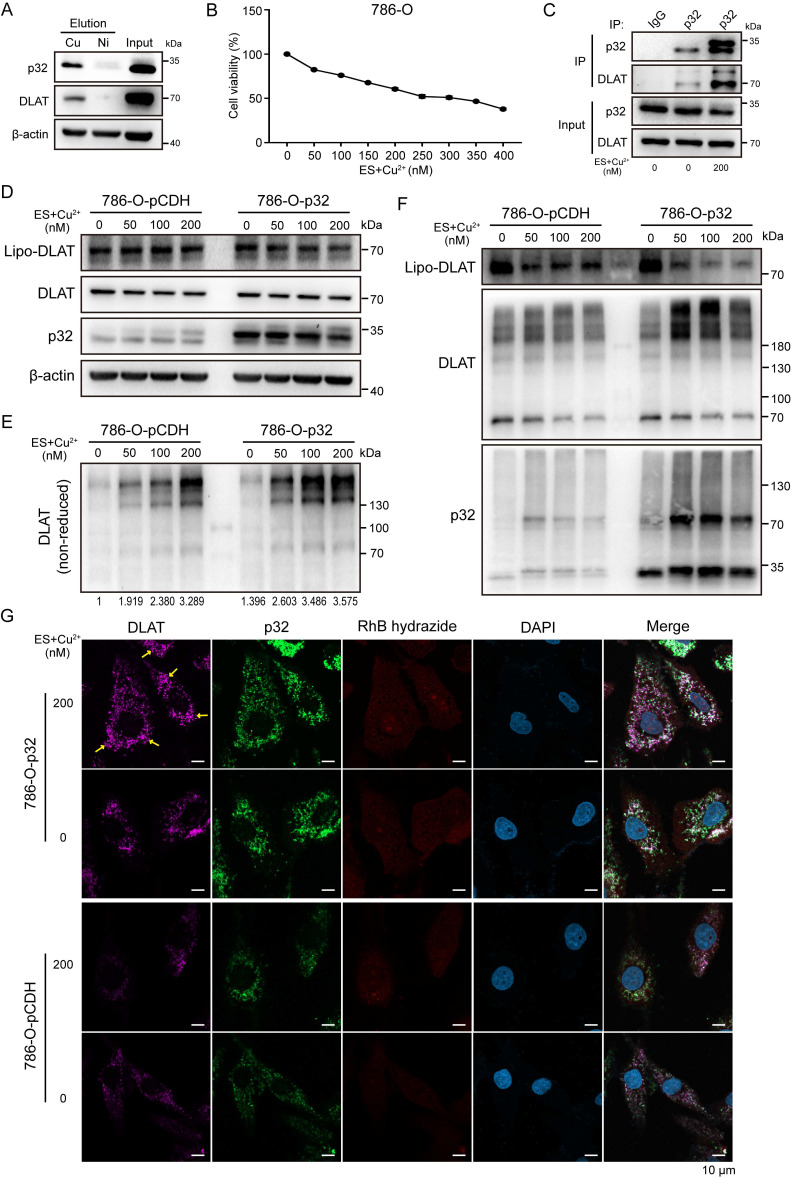
p32 promotes copper-induced oligomerization of lipo-DLAT in ccRCC cells. **(A)** Immunoblotting showed the binding of the 786-O cells' indicated proteins to copper (Cu) and nickel (Ni) of eluted proteins from the indicated metal-IDA beads. **(B)** Cell survival rate after treatment with 0-400 nM concentration gradient of elesclomol and CuSO_4_ in 786-O cells. **(C)** Western blot showed the co-immunoprecipitation of DLAT and p32 in 786-O cells after treating with or without 200nM elesclomol and CuSO_4_ for 24 h. 786-O-pCDH and 786-O-p32 cells were treated with concentration gradients of elesclomol and CuSO_4_, and the cellular proteins were extracted. **(D)** Western blotting showed the expression of the specified protein indicators after protein denaturation, **(E)** Non-reducing western blotting showed the expression of DLAT oligomers and **(F)** Western blotting showed the expression of the specified protein indicators after crosslinking protein. The relative quantification of the gray value of the bands was analysised with ImageJ software. **(G)** Immunofluorescence images showed the expression of indicated proteins after treating with or without 200nM elesclomol and CuSO_4_ for 24 h in 786-O-pCDH and 786-O-p32 cells. The yellow arrow pointed to the DLAT foci.

The study by Tsvetkov *et al.* demonstrated that excess copper binds and promotes the oligomerization of lipoylated DLAT. Hence, the study demonstrated that copper-induced cell death is mediated by the direct interaction of copper with lipoylated constituents of the TCA cycle 37. However, it remains unclear how these events may occur in ccRCC. Given our confirmation of p32 interaction with DLAT, we pursued the functional relationship by inducing excess copper. To test this, we measured copper affinity and found that both p32 and DLAT bound to Cu IDA beads but not Nickel in 786-O cells. This result suggests that p32 and DLAT could directly bind copper (Figure 6A). Copper ionophore elesclomol is known to shuttle copper into the cell 38. Hence, we investigated the impact of exogenous copper with elesclomol on cell viability. Notably, we observed 786-O cell viability decreased in a concentration-dependent manner when treated with copper and elesclomol (Figure 6B). Initially, to probe the cellular copper contents, ACHN and 786-O cells with or without copper were incubated with the labile copper sensing probe, the copper probe fluorescence signal was strongly detected in the copper treatment cells (Figure S3A). Interestingly, enhanced interaction between p32 and DLAT was observed in the copper treatment 786-O cells (Figure 6C). To investigate potential p32 function in lipoylation-dependent oligomerization of DLAT with copper, we examined lipoylation of DLAT by traditional gel electrophoresis and oligomerization of DLAT by non-reducing gel electrophoresis. As predicted, concomitant with the copper concentration increasing, we observed lipoylation of DLAT and total DLAT levels remained constant in p32 overexpression 786-O cells (Figure 6D). Moreover, p32 overexpression 786-O cells robustly increased levels of lipo-DLAT oligomers, whereas control cells mildly increased levels of lipo-DLAT oligomers (Figure 6E). In order to discuss whether p32 participates in oligomer composition, we applied protein cross-linking agent DSS to immobilize the protein polymer in cells for a moment, and then performed western blot analysis. We found that the position of DLAT lipoylation monomer decreased more significantly, while p32 appeared more obvious dimer bands in p32 overexpression 786-O cells (Figure 6F). This finding was confirmed with immunofluorescence, as we observed pronounced induction of DLAT foci by copper following elesclomol treatment, with foci also increased in p32 overexpressed 786-O cells (Figure 6G). We also observed similar result in ACHN cells. Stable expression of p32 significantly reduced DLAT lipoylation level but increased lipo-DLAT oligomerization (Figure S3B, C). Protein analysis after cross-linking also showed that DLAT lipoylation was reduced, and p32 was involved in oligomerization (Figure S3D). Additionally, we observed DLAT foci was robustly increased in copper treated p32 overexpressed ACHN cells (Figure S3E). These findings demonstrate that p32 promotes lipo-DLAT oligomerization in a copper-dependent manner and p32 may participate in the composition of oligomer.

### Combinational treatment of p32 and copper inhibits ccRCC progression through disruption of TCA cycle

**Figure 7:**
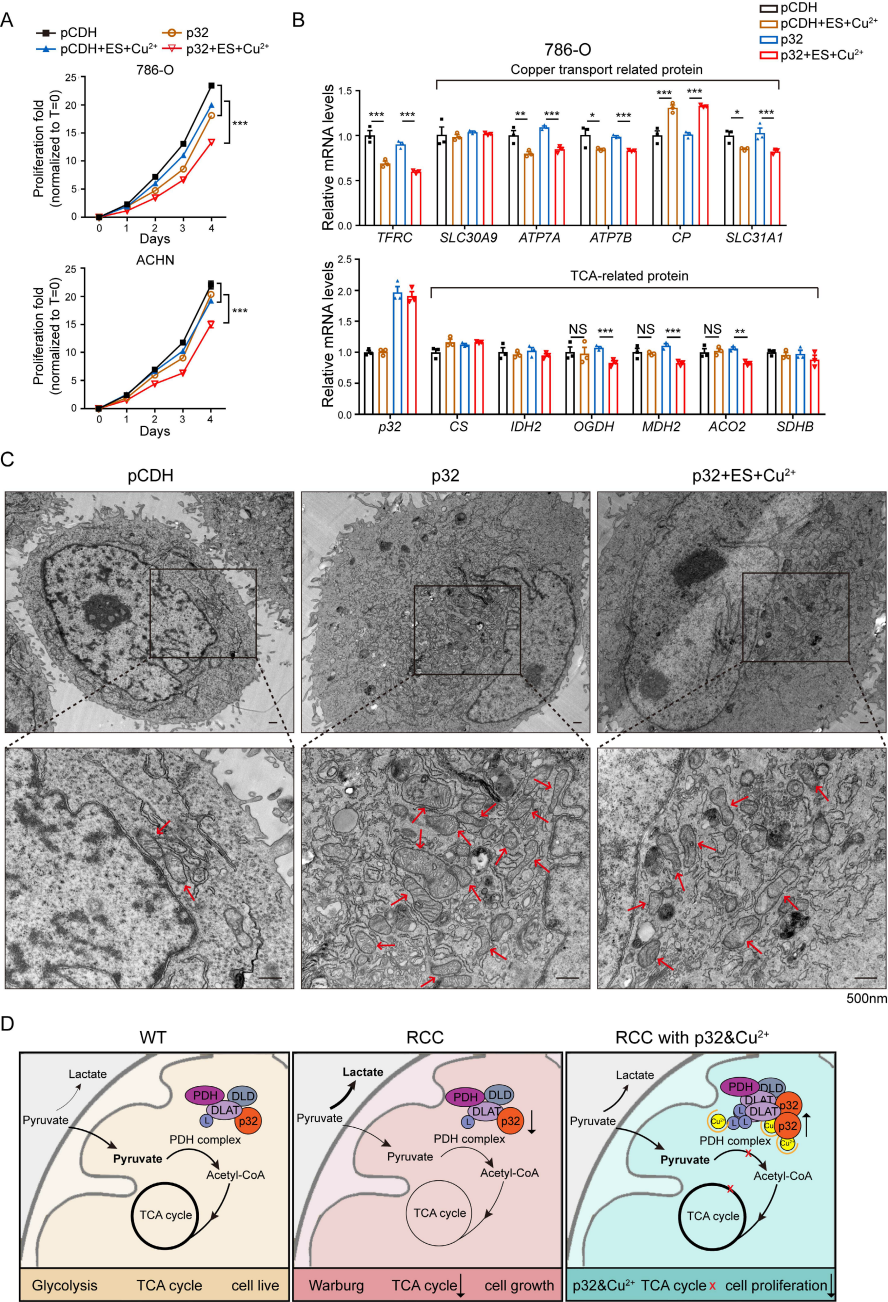
p32 promotes copper-induced TCA cycle disruption in ccRCC. **(A)** Cell proliferation after treating 786-O cells with or without 200 nM elesclomol and CuSO_4_ or treating ACHN cells with or without 400 nM elesclomol and CuSO_4_ (n=4). **(B)** Quantitative RT-PCR analysis measured *TFRC, SLC30A9, ATP7A, ATP7B, CP* and *SLC31A1* relative mRNA levels (upper panel) and *CS, IDH2, OGDH, MDH2, ACO2* and *SDHB* relative mRNA levels (lower panel) in 786-O cells with or without treatment of 200 nM elesclomol and CuSO_4_ (n=3). **(C)** The representative pictures of the morphology of mitochondria in cells under the transmission electron microscope. Higher magnification views of the indicated areas are shown on the bottom.The red arrows refer to the mitochondria. **(D)** The mechanism of p32 promoting copper-induced DLAT lipoylation oligomerization, thereby affecting TCA cycle to inhibit the proliferation of ccRCC. The results were the mean ± SEM of three independent experiments., *P*-values were determined by unpaired two-tailed Student's *t*-test. Statistically significant differences were indicated: *P<0.05, **P<0.01, ***P<0.001.

There are ongoing efforts to explore the utility of copper in cancer treatment. For example, elesclomol, a copper-binding small molecule, has undergone clinical trials for the treatment of fallopian tube, ovarian, or primary peritoneal cancer 39. To investigate whether p32 chelation by copper contributes to its antitumor function, the proliferation of p32 overexpression with copper and elesclomol treatment in 786-O and ACHN cells was analyzed. It found that p32 overexpression combination treatment with copper and elesclomol dramatically reduced cell viability both in ACHN and 786-O cells (Figure 7A). The proliferation studies showed us the role of combinational treatment of p32 and copper on the TCA cycle enzymes expression. There was a significant decrease in mRNA expression of TCA cycle enzymes in p32 and copper treatment ccRCC cells, including *ACO2, OGDH*, and *DMH2*, relative to other three (Figure 7B). Furthermore, copper-related mRNA levels were measured. *ATP7A, ATP7B, SLC31A1* and *TFRC* (Fe-related) were significantly reduced, and *CP* was increased after copper treatment in both the control and p32 overexpresed cells (Figure 7B). PDH activity was diminished in p32 overexpressing cells within gradient copper and elesclomol stimulation (Figure S2C). The place where the TCA cycle occurs is the mitochondria. In order to explore whether the morphology of mitochondria will be affected, we made cell sections and carried out transmission electron microscope experiments to observe the morphology of mitochondria. The results indicated that the number of mitochondria in p32 overexpression cells was significantly increased with normal morphology; while the morphology of mitochondria was significantly abnormal after treating with copper, the mitochondria became round and swollen, and the internal crista structure was significantly disordered, indicating that the function of mitochondria was damaged (Figure 7C). Taken together, these evidences indicate that the combinational treatment of p32 and copper inhibits ccRCC progression through disruption of TCA cycle and mitochondrial damage (Figure 7D).

## Discussion

The most lethal form of kidney cancer is metastatic renal cell carcinoma (mRCC). Immunocheckpoint inhibitors (ICIs), mTOR inhibitors, vascular endothelial growth factor receptor tyrosine kinase inhibitors (VEGFR TKIs), and other medications are examples of systemic therapeutic options 40, 41. Further investigation into the molecular underpinnings of RCC development has been spurred by the insensitivity to chemotherapy and radiation treatment, as well as the severe side effects of targeted therapy. Thus, new targeted anticancer medications have also been aided by this 42. Most occurrences of ccRCC are associated with the inactivation of the von Hippel-Lindau tumor-suppressor gene (VHL).

For mRCC, the VHL-HIF-VEGF/VEGFR pathway is a well-researched therapeutic target 43. Three major metabolic abnormalities were found at the proteome level, including energy metabolism, lipid metabolism, and one-carbon metabolism, in 232 pairs of Chinese patient tissue samples close to the tumor and ccRCC tumor 44. Because it affects several metabolic pathways, such as glycolysis, the TCA cycle, and the metabolism of glutamine, fatty acids, and amino acids, renal cancer has been categorized as a "metabolic disease" 45-47. Significant metabolic reprogramming was found in research involving around 500 ccRCC samples, which led to poor survival and prognosis results in RCC. The primary genes that control the process of metabolic reprogramming in renal cancer are Myc, VHL, PTEN, Akt, mTOR, and TSC1/2 48-52. The primary energy source in biological systems is glucose. Oxidative stress and cellular cytotoxicity are caused when cancer cells are deprived of glucose 53. In terms of glucose excretion, gluconeogenesis, and glucose reabsorption, the kidneys are essential to glucose homeostasis 54. Courtney et al. used isotope labeling methods to show that, in comparison to tumors found in other anatomic regions, ccRCC cells display increased glycolysis, reduced pyruvate dehydrogenase flux, and impaired TCA cycle activity 55. One possible strategy to slow the growth of tumors, including kidney cancer, is to restrict the glucose transport in cancer cells. STF-31, a GLUT1 inhibitor, is specifically designed to target RCC cells in order to efficiently hinder tumor development and induce cell death 56. Compared to other tumor forms, renal cancer is more sensitive to changes in glucose metabolism, which can have a substantial effect on the tumor's ability to spread.

Many lines of evidence suggest that RCC also exhibits changes in mitochondrial metabolism, despite the increased emphasis on the expression of glycolytic enzymes in tumor metabolism 57-59. In a preclinical orthotopic model of RCC, for example, it has been demonstrated that the administration of pharmacological inhibitors that target the transforming growth factor-beta (TGF-β) pathway efficiently restores the expression levels of TCA cycle enzymes 60. Furthermore, in the setting of ccRCC, a positive feedforward loop between glycerol-3-phosphate dehydrogenase 1 (GPD1) and hypoxia-inducible factor 1-alpha (HIF1α) has been clarified. Particularly in ccRCC cells, this regulatory loop works to inhibit lipid metabolism and reduce mitochondrial activity 61. HIFs have been found to be regulators that, by transcriptionally activating PDK1, block the conversion of pyruvate to acetyl-CoA and decrease the metabolic flow towards the TCA cycle 62, 63. Mutations in the relevant enzymes commonly cause the TCA cycle to be disturbed in cancer cells. Normal cells can become cancerous by mutations in the genes encoding fumarate hydratase (FH), succinate dehydrogenase (SDH), and isocitrate dehydrogenase (IDH) 64-69. As tumor suppressors, SDH and FH are mutated to lose their enzymatic activity. This results in a buildup of fumarate and succinate, which can promote the formation of tumors. Moreover, an examination of data from The Cancer Genome Atlas (TCGA) has shown a correlation between poor patient outcomes and the downregulation of TCA cycle enzyme expression 70. According to these results, RCC advancement is strongly linked to mitochondrial metabolism, suggesting that blocking mitochondrial metabolism might be a novel target for the therapy of ccRCC.

Numerous cellular processes, including as immunological response, inflammation, apoptosis, splicing, mitochondrial metabolism and dynamics, and regulation of several cell signaling pathways, depend critically on p32 21. Reduced patient survival is linked to higher expression of p32, which is often elevated in epithelial malignancies. On the other hand, p32's precise roles can change based on the kind of cancer. Overexpression of p32 stimulates the AKT-dependent pathway in HepG2 cells to upregulate cyclin D1 and encourage cell proliferation 71. According to Sinha S. et al., p32 is essential for controlling melanoma cell invasion, migration, and proliferation. They discovered that p32 accomplishes this via modifying the expression of oncogenes and markers linked to the epithelial to mesenchymal transition (EMT) in both human and animal melanoma 72. Furthermore, through its association with integrin αvβ3, p32 was discovered to stimulate NF-kappa B dependent activation of MMP-2. Tumor growth and cell migration are influenced by this regulatory mechanism 73. The hypoxia-induced activation of the PKC/NF-KB/VCAM-1 signaling pathway in TNBC is inhibited by p32 deletion. The result of this inhibition is the prevention of cancer cell metastasis 74. Through its association with p32, circular RNA MTCL1 has been shown to exert regulatory control over the Wnt/β-catenin pathway, hence modulating the course of laryngeal squamous cell carcinoma 75. Furthermore, through the IGF-1/IGF-1R signaling pathway, p32 mediates pancreatic cancer's hepatic metastasis 76. Recent data indicates that p32 may be used by tumor cells to control the ratio of glycolysis to OXPHOS 23. Reduced mitochondrial respiration and lipid consumption, heightened susceptibility to mitochondrial stress, and a metabolic switch from OXPHOS to glycolysis are all caused by p32 loss. These alterations are linked to the suppression of 3D spheroid formation and cancer cell apoptosis 77. Our earlier studies have shown that p32 may control XDH-mediated reactive ROS generation, which in turn can improve the breakdown of hypoxanthine and accelerate apoptosis in RCC cells 30. It has been determined that p32 controls the GSK3/β-Catenin/L1CAM signaling pathway, which in turn controls the metastasis of RCC 25. Additionally, p32 interacts with YBX1 to modify YBX1 phosphorylation and nuclear translocation, which inhibits YBX1 activation. Through modification of the AR-mediated MMP9 signals, this interaction suppresses the migration and invasion of RCC cells 32. Research has progressed to the point where p32 is understood to be a multi-compartment, multifunctional protein 78. P32 is present in a number of cellular locations, most notably the mitochondrial matrix and extracellular cell surface 79. A number of signaling pathways, including the TCA cycle, OXPHOS, and FAO, are centered on mitochondria. Cells can respond and adapt to a range of stressful situations thanks to these pathways 80, 81. Therefore, the purpose of this study is to explore the critical function of p32 in RCC mitochondria, which may work in concert with other mechanisms to obstruct RCC advancement or serve as a critical modulatory mechanism.

PDH serves as a crucial metabolic hub that facilitates the oxidation of pyruvate following glycolysis. It supplies the TCA cycle with substrates to meet cellular energy requirements. The regulation of PDH activity is primarily controlled by post-translational modifications of its subunits, notably phosphorylation and dephosphorylation of the PDHA1 subunit, as well as lipoylation of the DLAT subunit. These modifications play a significant role in fine-tuning PDH function 26, 28, 82, 83. DLAT upregulation has been observed in gastric cancer cells, while a novel mechanism reveals that PM2.5 promotes glycolysis in NSCLC cells through Sp1-mediated transcriptional regulation of DLAT 84, 85. We characterized the interaction between p32 and DLAT in mitochondria through mass spectrometry analysis, and revealed their correlation in ccRCC. Interestingly, in the current study, we observed no functional role of p32 in the expression of DLAT, but p32 regulated PDH activity. Simultaneously, as a physical interaction between p32 and DLAT, p32 displayed marginal regulatory role towards lipo-DLAT. Even so, we tested if aggravated OXPHOS by p32 overexpression can be reversed by simultaneous reduction of DLAT levels in ccRCC cells. As expected, deletion of DLAT relieved excessive OXPHOS and suppressed proliferation and migration induced by p32. Finally, we have demonstrated the binding of DLAT and p32 C-terminal peptide, providing molecular insights about the role of DLAT in p32 regulation of ccRCC.

*Tsvetkov et al.* found that copper directly binds and promotes the oligomerization of lipo-DLAT. Combined with our research, it causes us to think: will p32 participate in it? Our findings demonstrated that p32 directly binds copper and DLAT. How p32/copper-mediating DLAT post-translational modification changes in ccRCC cells alter the mitochondrial metabolism and TCA cycle, and whether p32/copper combinational treatment can be used as drugs in cancer therapy with low adverse effects remain unsolved problems. We have addressed these questions by demonstrating that p32 inhibits ccRCC progression through oligomerization of lipo-DLAT and disruption of TCA cycle in copper-dependent condition. In the absence of p32, mitochondria exhibited fragmented shape but lacked cristae structure 86, 87, we found that number of mitochondria was significantly increased in cells overexpressing p32, suggesting that p32 exerts regulatory control over both the morphology and function of mitochondria. Mitochondria from p32/copper combinational treatment cells displayed enlarged intermembrane spaces and structural damage, as previously described in patients and mouse models 88, 89. We postulate that p32 induces disruption of specific mitochondrial metabolic enzymes that triggers a decrease copper-induced cell proliferation. Understanding p32/copper's role in glycolysis metabolism could pave the way for concomitant drugs to be utilized as anti-cancer pharmaceuticals or adjunctive therapies.

The identification of p32 as a central player in mitochondrial metabolism, along with the potential therapeutic benefits of combinational treatment using copper and elesclomol, presents a promising and pharmacologically feasible strategy for antitumor therapies. Notably, several extensively studied copper-based pharmacological agents are currently available, many of which could be considered as viable drug options for phase II and III clinical trials in cancer therapy 90, 91. Most of the studies focus on copper depletion in tumor environment to reduce angiogenesis 92-94. Therefore, our speculation suggests that copper therapy may exhibit enhanced efficacy in renal cancer patients with high p32 expression. Alternatively, combining copper therapy with interventions aimed at inducing upregulation of p32 in renal cancer tissue could offer potential clinical applications and value. However, copper acts actively and high levels could present deleterious toxicity concerns. Concomitant antitumor therapies are encouraged to effectively maintain the metabolic profile sensitivity.

## Conclusions

In this study, we have provided evidence of tissue-specific expression of p32 in ccRCC and its inhibitory effect on ccRCC progression *in vivo*. We found p32 promotes the OXPHOS of ccRCC by combining with DLAT in cells, a finding that we validated in clinical samples. Finally, we report that p32 enhances copper-induced lipo-DLAT oligomerization and TCA cycle damage, which subsequently inhibits the proliferation of ccRCC.

## Supplementary Material

Supplementary figures and tables.

## Figures and Tables

**Table 1:** Clinical relevance of p32 and DLAT in ccRCC by western blot

		p32 expression		Spearman's r Correlation coefficent	*P* value
		Up	Down		
DLAT	Up	4	1	0.599	<0.001***
	Down	3	22		

Note: The expression of p32 and DLAT was quantified and compared between ccRCC tumor (T) and paired para-tumor tissue (P). The relative ratio T/P > 1 was defined as “Up”, and T/P < 1 was defined as “Down”. Spearman test were used. The results were considered statistically significant at *P* < 0.05. ***P < 0.001.

**Table 2:** Clinical relevance of p32 and DLAT in ccRCC by IHC

		p32 expression		Spearman's r Correlation coefficent	*P* value
		positive	negative		
DLAT	positive	9	3	0.477	<0.001***
	negative	11	52		

Note: IHC detected the expression of protein p32 and DLAT in 75 ccRCC tissues. Spearman test were used. The results were considered statistically significant at *P* < 0.05. ***P < 0.001.

**Table 3:** Correlation between expression of p32 and DLAT and clinicopathologic characteristics in ccRCC patients

		N	DLAT expression		*p* value	p32 expression		*p* value
			-	+		-	+	
Gender	Male	54	44	10	0.34	37	17	0.131
	Female	21	19	2		18	3	
Age	<60	30	23	7	0.157	20	10	0.286
	≥60	45	40	5		35	10	
Tumor size	<4	16	15	1	0.459	11	5	0.895
	>4,≤7	24	20	4		18	6	
	>7	35	28	7		26	9	
T stage	T1-2	46	37	9	0.289	28	18	0.002**
	T3-4	29	26	3		27	2	
Fuhrman grade	I,II	39	31	8	0.267	24	15	0.016*
	III,IV	36	32	4		31	5	
Location	Tumor	75	63	12	<0.001***	55	20	<0.001***
	Para-tumor	75	11	64		10	65	

Note: IHC staining results (+): positive, (-): negative. Pearson's *X*^2^ tests were used. The results were considered statistically significant at *P* < 0.05. *P < 0.05, **P < 0.01, ***P < 0.001.

## Abbreviations

RCC: Renal cell carcinoma
ccRCC: Clear cell renal cell carcinoma
TCA cycle: Tricarboxylic acid cycle
PDHc: Pyruvate dehydrogenase complex
H&E: Hematoxylin and eosin
PFA: Paraformaldehyde
ECAR: Extracellular acidification rate
OCR: Oxygen consumption rate
2-DG: 2-deoxy glucose
CCK-8: Cell Counting Kit-8
DSS: Disuccinimidyl suberate
OXPHOS: Oxidative phosphorylation
C1QBP: Complement C1q binding protein
HAPB1: Hyaluronic acid-binding protein
gC1qR: Receptor for globular head domains of complement 1q
DLAT: Dihydrolipoyl transacetylase
DLD: Dihydrolipoyl dehydrogenase
IP: Immunoprecipitation
STRING: Search Tool for the Retrieval of Interacting Genes
KIRC: Kidney renal clear cell carcinoma
IHC: Immunohistochemistry
mRCC: Metastatic RCC
ICIs: Immune-checkpoint inhibitors
VEGFR: Vascular endothelial growth factor receptor
TKIs: Tyrosine kinase inhibitors
VHL: Von Hippel-Lindau
HIF: Hypoxia-inducible factor
