# Supplementary material for: p32 regulates glycometabolism and TCA cycle to inhibit ccRCC progression via copper-induced DLAT lipoylation oligomerization
Source: Int J Biol Sci. 2024 Jan 1;20(2):516–36. doi: 10.7150/ijbs.84399 (PMC10758103; doi:10.7150/ijbs.84399)
Supplement: Supplementary file 1 — Supplementary figures and tables. [file ijbsv20p0516s1.pdf]

Supplementary Information for

p32 regulates glycometabolism and TCA cycle to inhibit ccRCC progression via copper-induced DLAT lipoylation oligomerization

This file includes:

Figures S1-S3 and legends

- ✓ Figure S1. p32 expression and the relationship between p32, DLAT and clinical parameters in the database.
- ✓ Figure S2. The C-terminal deletion of p32 does not alter the glucose metabolism phenotype of ccRCC.
- ✓ Figure S3. p32 promotes copper-induced oligomerization of lipo-DLAT in ACHN cells.

Supplementary Tables S1-S3

- ✓ Table S1. The sequences of DLAT siRNAs
- ✓ Table S2. The sequence of primer (F: Forward primer; R: Reverse primer)
- ✓ Table S3. The basic situation of selected patients in the TCGA datasets

Supplementary Figures

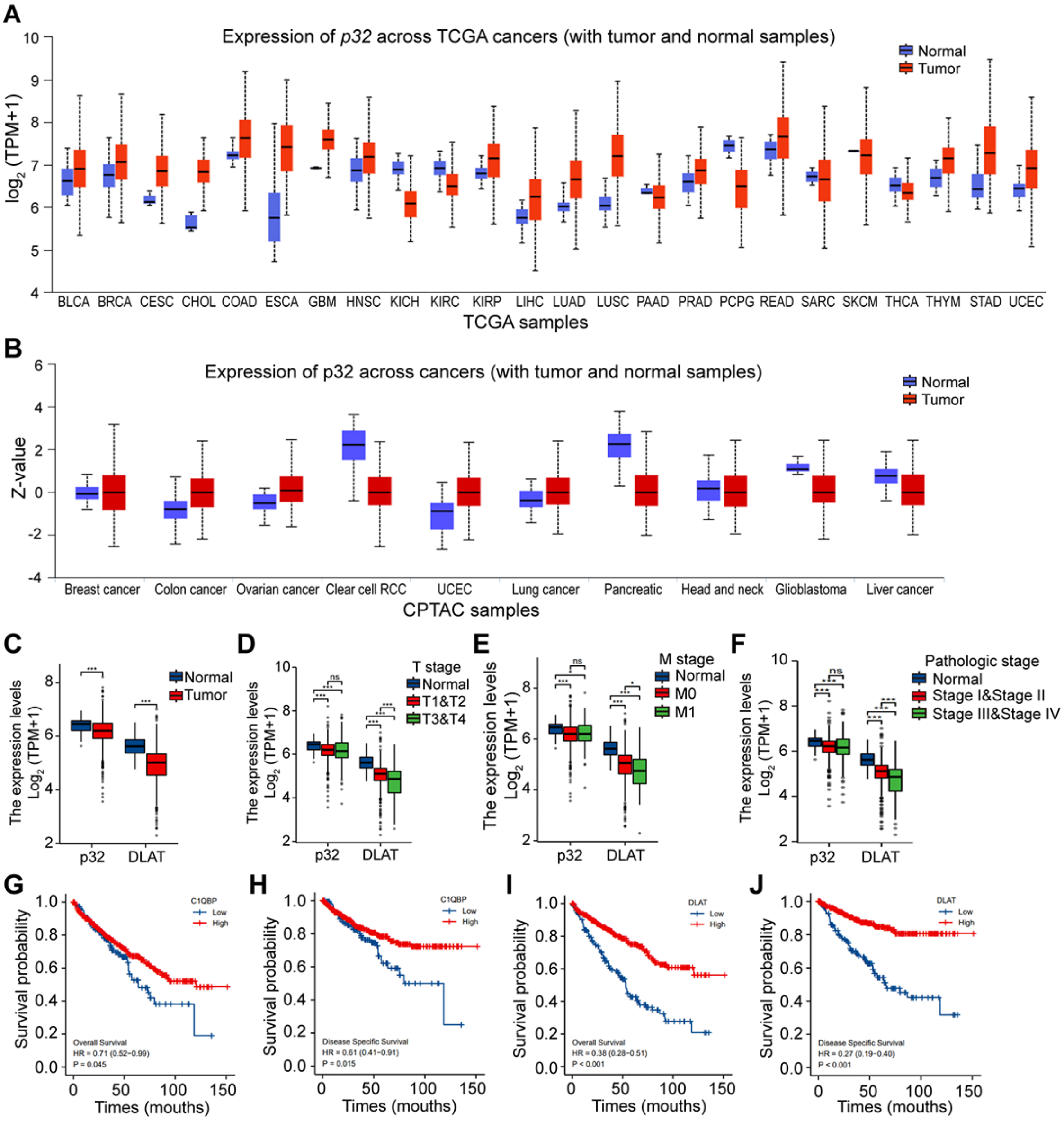

Figure S1. p32 expression and the relationship between p32, DLAT and clinical parameters in the database. **(A)** Expression levels of *p32* mRNA in pan-cancer (http://ualcan.path.uab.edu/). **(B)** Protein expression levels of p32 in pan-cancer (http://ualcan.path.uab.edu/). The correlation between p32, DLAT with **(C)** normal and tumor, **(D)** T stage, **(E)** M stage, **(F)** pathological stage (normol n=72, tumor n=539). The effects of p32 expression on **(G)** overall survival (OS) time, **(H)** disease-specific survival (DSS) time in patients with KIRC were analyzed by survival curve (n=539, OS P=0.045, DSS P=0.015). The effects of DLAT expression on **(I)** OS time, **(J)** DSS time in patients with KIRC were analyzed by survival curve (n=539, OS P<0.001, DSS P<0.001). Statistically significant differences were indicated: *P<0.05, **P<0.01, ***P<0.001. NS: no significant difference.

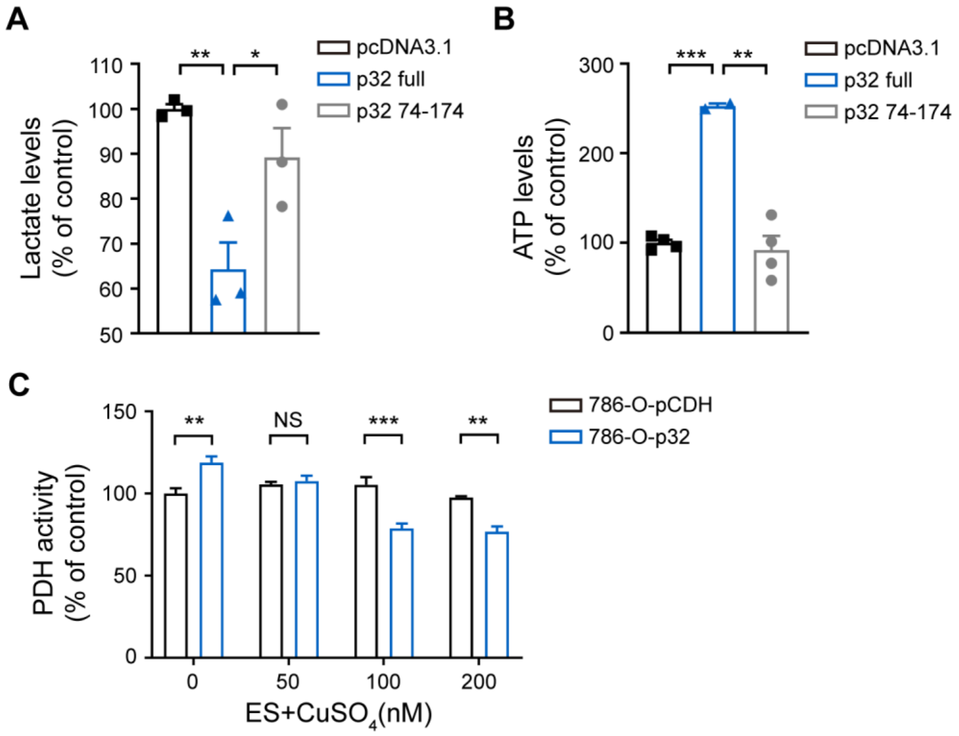

Figure S2. The C-terminal deletion of p32 does not alter the glucose metabolism phenotype of ccRCC. 786-O cells were transfected with p32-full and p32-74-174, **(A)** Lactate levels and **(B)** ATP levels were examined. **(C)** PDH activity were examined in p32 overexpression 786-O cells within gradient copper and elesclomol stimulation.

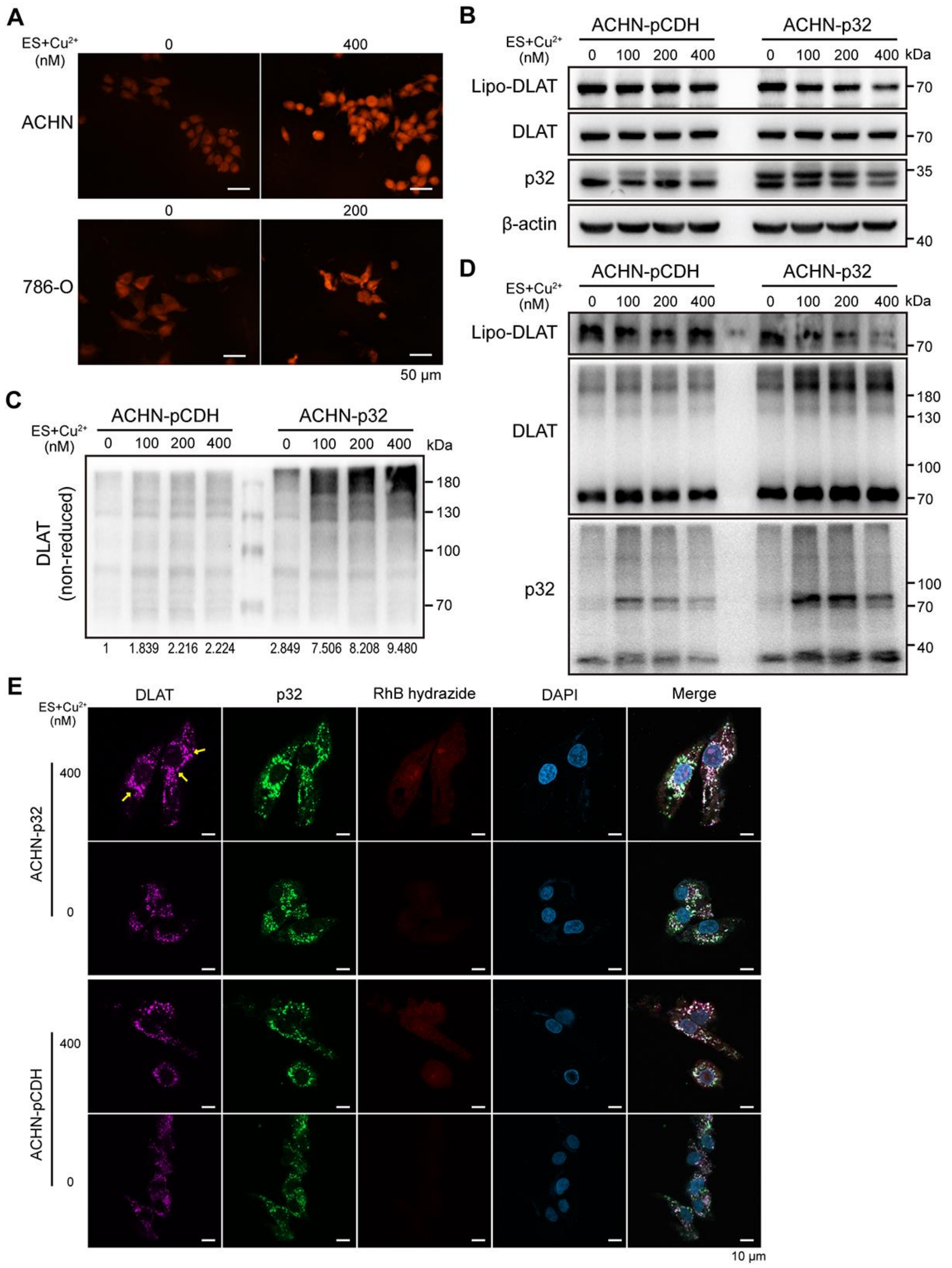

Figure S3. p32 promotes copper-induced oligomerization of lipo-DLAT in ACHN cells. **(A)** ACHN and 786-O cells were treated with or without elesclomol and $CuSO_4$ at a suitable drug concentration that did not affect cell morphology for 24 h, and then the intracellular copper ion content was observed under a microscope by adding rhodamine B hydrazide probe. ACHN-pCDH and ACHN-p32 cells were treated with concentration gradients of elesclomol and $CuSO_4$, and the cellular proteins were extracted. **(B)** Western blotting showed the expression of the specified protein

indicators after protein denaturation, **(C)** Non-reducing western blotting showed the expression of DLAT oligomers and **(D)** Western blotting showed the expression of the specified protein indicators after crosslinking protein. The relative quantification of the gray value of the bands was analysised with ImageJ software. **(E)** Immunofluorescence images showing the expression of indicated proteins after treating with or without 400 nM elesclomol and $CuSO_4$ for 24 h in ACHN-pCDH and ACHN-p32 cells. The yellow arrow points to the DLAT foci.

Supplementary Tables

Table S1. The sequences of DLAT siRNAs

Name	Target sequence
si-*DLAT*#1	CCACTCTGTATCATTGTAGAA
si-*DLAT*#2	GCTGAGTTTAGAAAGTACCTT
si-*DLAT*#3	CCGCATCAGAAGGTTCCATTA

Table S2. The sequence of primer (F: Forward primer; R: Reverse primer)

Genes	Primers sequences (5′ to 3′)
GAPDH	F: TGCACCACCAACTGCTTAGC
	R: GGCATGGACTGTGGTCATGAG
p32	F: TTTGATGGTGAGGAGGAACC
	R: GCCTTCTTGCCATCATCATT
DLAT	F: CAGGGTGGCACTTTTACGAT
	R: GAAGCACCAATTGCCAAAAT
LDHA	F: TGTGCCTGTATGGAGTGGAA
	R: AGCACTCTCAACCACCTGCT
PKM2	F: CTATCCTCTGGAGGCTGTGC
	R: GAGGCTCGCACAAGTTCTTC
TFRC	F: AAAATCCGGTGTAGGCACAG
	R: CACCAACCGATCCAAAGTCT
SLC30A9	F: GTCATGGGATTGCTTCATCC
	R: ATTCCTTTAGCCCGAGCATT
ATP7A	F: CTGGCAAGGCAGAAGTAAGG
	R: TTCCCCTCACAACAAGTTCC
ATP7B	F: AAGTCCCCACAATCAACCAG
	R: ACCAACACGGAGAGAACACC

CP	F: ATCCGTGGGAAGCATGTTAG
	R: TGAGTCACTTCCAGGTGCTG
SLC31A1	F: CAGCATTCGCTACAATTCCA
	R: GGTGAGGAAAGCTCAGCATC
CS	F: GCAGAAGGAAGTTGGCAAAG
	R: CGCGGATCAGTCTTCCTTAG
IDH2	F: CTCATCAGGTTTGCCCAGAT
	R: AGGAAGTCCGTGGTGTTCAG
OGDH	F: GGAATCAGCACTTCCTCTGC
	R: CAGGGGTCTCAAACTTCTGC
MDH2	F: GCAGCCACTTTCACTTCTCC
	R: GCTGTTCAGGTCCGAGGTAG
ACO2	F: GTCACGTCCCCAGAGATTGT
	R: CCTCCAGCCTGAACTTCTTG
SDHB	F: GGAAGGCAAGCAGCAGTATC
	R: ATTTGTCTCCGTTCCACCAG

Table S3. The basic situation of selected patients in the TCGA datasets

**Characteristics**	**Total (N)**	**Unavailable number (N)**
**Age**	**539**	**0**
<=60	269	
>60	270	
**Gender**	**539**	**0**
Female	186	
Male	353	
**Race**	**532**	**7**
Asian	8	
Black or African [illegible]	57	

White	467	
**T stage**	**539**	**0**
T1	278	
T2	71	
T3	179	
T4	11	
**N stage**	**257**	**282**
N0	241	
N1	16	
**M stage**	**506**	**33**
M0	428	
M1	78	
**Pathologic stage**	**536**	**3**
Stage I	272	
Stage II	59	
Stage III	123	
Stage IV	82	
**Histologic grade**	**531**	**8**
G1	14	
G2	235	
G3	207	
G4	75	
